# Particle number analysis of lipoprotein subclasses by gel permeation HPLC in patients with cholesteryl ester transfer protein deficiency

**DOI:** 10.1371/journal.pone.0190875

**Published:** 2018-01-05

**Authors:** Takeshi Okada, Tohru Ohama, Mitsuyo Okazaki, Kotaro Kanno, Hibiki Matsuda, Masami Sairyo, Yinghong Zhu, Ayami Saga, Takuya Kobayashi, Daisaku Masuda, Masahiro Koseki, Makoto Nishida, Yasushi Sakata, Shizuya Yamashita

**Affiliations:** 1 Department of Cardiovascular Medicine, Osaka University Graduate School of Medicine, Osaka, Japan; 2 Department of Dental Anesthesiology, Osaka University Graduate School of Dentistry, Osaka, Japan; 3 Tokyo Medical and Dental University, Tokyo, Japan; 4 Health Care Division, Health and Counseling Center, Osaka University, Osaka, Japan; 5 Department of Community Medicine, Osaka University Graduate School of Medicine, Osaka, Japan; 6 Department of Cardiology, Rinku General Medical Center, Osaka, Japan; University of Basque Country, SPAIN

## Abstract

**Objective:**

We previously reported that patients with cholesteryl ester transfer protein (CETP) deficiency (CETP-D) have a higher prevalence of atherosclerotic cardiovascular disease, in spite of increased HDL-C levels. However, characterization of HDL in CETP-D has not been well described. Therefore, we examined HDL particle number (PN) rather than HDL-C level.

**Approach and results:**

Nine patients with CETP-D and 9 normolipidemic subjects were enrolled. We performed gel permeation high-performance liquid chromatography (GP-HPLC) analysis, determined the cholesterol and triglyceride composition of all lipoprotein subclasses, and calculated the PN of each subclass, which consisted of 3 VLDL (large, medium, and small), 4 LDL (large, medium, small, and very small), and 5 HDL (very large, large, medium, small, and very small) subclasses. The PNs of large and medium LDL were significantly lower in CETP-D than that in healthy subjects (0.66- and 0.63-fold decrease, respectively; p<0.001), whereas the PN of very small LDL, which is known to be atherogenic, was significantly higher (1.36-fold increase, p = 0.016). The PNs of very large and large HDL in CETP-D were markedly higher than that in healthy subjects (19.9- and 4.5-fold increase, respectively; p<0.001), whereas the PNs of small and very small HDL, which have more potent anti-atherogenic functions, were significantly lower (0.76- and 0.61-fold decrease, respectively; p<0.001).

**Conclusion:**

We have assessed the PNs of detailed subclasses of patients with CETP-D for the first time. The PN of larger HDL was markedly increased, that of smaller HDL was decreased, and that of very small LDL was increased, suggesting that CETP-D has pro-atherogenic lipoprotein properties.

## Introduction

Epidemiological studies have shown that high LDL-cholesterol (LDL-C) and low HDL-cholesterol (HDL-C) levels are positively and independently correlated with cardiovascular disease (CVD) events[1]. Randomized controlled trials have revealed that LDL-C lowering drugs, such as statins, are effective both for primary[2] and secondary[3] prevention. By contrast, drugs that increase HDL-C levels, such as niacin[4] and most cholesteryl ester transfer protein (CETP) inhibitors, have been unable to reduce CVD events. Studies of the CETP inhibitors torcetrapib[5], dalcetrapib[6], and evacetrapib[7] were terminated because of increased mortality or no significant effects on major CVD events, although these drugs increased HDL-C or even decreased LDL-C levels. Anacetrapib has been reported to show a modest reduction of coronary events[8], but the drug development has been discontinued recently. We previously reported that patients with hyperalphalipoproteinemia (HALP) caused by CETP deficiency (CETP-D), who also showed similar lipid profiles to CETP inhibitor-treated patients, were not protected against CVD[9]. Thus, we hypothesized that inhibition or deficiency of CETP might affect the structural alterations of lipoproteins, including lipoprotein particle distribution or lipoprotein particle numbers (PNs).

To test this hypothesis, we performed gel permeation high-performance liquid chromatography (GP-HPLC) analysis, which can directly determine the cholesterol (Cho) or triglyceride (TG) composition of major lipoproteins or their subclasses. We then analyzed the lipoproteins according to their particle size. Furthermore, we have recently developed a novel strategy to calculate the PN of each lipoprotein subclass by GP-HPLC[10]. In the current study, we applied this method to identify the lipoprotein abnormalities in more detail in patients with CETP-D to determine whether CETP-D is atherogenic.

## Materials and methods

### Subjects

We enrolled 9 patients with homozygous CETP-D whose serum CETP mass was <0.1 μg/mL as well as 9 age- and sex-matched healthy normolipidemic subjects. The investigation conformed to the principles outlined in the Declaration of Helsinki. All subjects provided written informed consent, and the research protocol was approved by the Ethics Committee of Osaka University Hospital. Venous blood was drawn after overnight fasting for 12 h. Serum was separated by low-speed centrifugation (3000 rpm, 15 min, 4°C) and aliquots were immediately frozen at -80°C until use.

### Serum lipids and lipoproteins

Serum total cholesterol (TC), TG, HDL-C, and LDL-C levels were determined by enzymatic methods (Sekisui Medical Co., Tokyo, Japan). Serum concentrations of apoA-I, A-II, B, C-II, C-III, and E were measured by an immunoturbidimetric method (Sekisui Medical Co., Tokyo, Japan). Serum CETP mass levels were determined by an enzyme immunoassay (BML INC., Tokyo, Japan).

### Calculation of lipoprotein particle number by GP-HPLC

Serum lipoproteins were analyzed using the GP-HPLC system as previously described [11], and recently reviewed[12]. Briefly, lipoproteins in fresh-frozen serum (4 μl) were separated with tandemly connected Skylight PakLP1-AA gel permeation columns (Skylight Biotech Inc., Akita, Japan, 300 mm × 4.6 mm I.D.). The column effluent was then equally split into two lines by a micro splitter, and each effluent was allowed to react at 37°C with the Cho and TG reagents. Absorbance at 550 nm was continuously monitored after each enzymatic reaction in two reactor coils (PTFE; 25 m × 0.18 mm I.D.). The particle size of each lipoprotein was determined by the retention time of each peak observed on a chromatogram using a linear calibration curve. The Cho and TG levels of the 20 subclasses were defined by component peak analyses on the basis of lipoprotein particle size with the Gaussian curve fitting technique[13][14]. The following definitions of lipoprotein particle sizes for the 20 lipoprotein subclasses were used:

CM: CM1 and CM2, >90 nm and 75 nm in diameter, respectively. VLDL subclasses: large VLDL (VLDL1, VLDL2 and VLDL3, 64, 53.6 and 44.5 nm in diameter, respectively), medium VLDL (VLDL4, 36.8 nm in diameter), and small VLDL (VLDL5, 31.3 nm in diameter). LDL subclasses: large LDL (LDL1, 28.6 nm in diameter), medium LDL (LDL2, 25.5 nm in diameter), small LDL (LDL3, 23.0 nm in diameter) and very small LDL (LDL4, LDL5 and LDL6, 20.7, 18.6 and 16.7 nm in diameter, respectively). HDL subclasses: very large HDL (HDL1 and HDL2, 15.0 and 13.5 nm in diameter, respectively), large HDL (HDL3, 12.1 nm in diameter), medium HDL (HDL4, 10.9 nm in diameter), small HDL (HDL5, 9.8 nm in diameter) and very small HDL (HDL6 and HDL7, 8.8 and 7.6 nm in diameter, respectively). The average particle diameters of the major subclasses of lipoproteins were obtained by calculating the weighted average of each fraction.

The particle number (PN) of the lipoprotein subclasses was calculated using a “spherical particle model”. As we recently reported[10], the particle size of lipoproteins belonging to a certain subclass is assumed to be almost constant. We focused on the sum volume of cholesteryl ester (CE) and TG within the core of lipoprotein subclasses. The sum volume of CE and TG in a certain subclass can be calculated from the amounts of Cho and TG without free cholesterol (FC) measurement. This calculation is based on the assumption that the ratio of FC to (CE + TG) in a certain subclass is almost constant, which we determined in the general cohort[10]. However, the fraction of HDL7, which contains pre-beta-1 HDL, cannot be applied to this spherical particle model because pre-beta-1 HDL consists of phospholipids, apoA-I and FC, with no core components[10], so the PN of very small HDL was assessed only by the PN of HDL6 in this study. The PN belonging to a certain subclass can be calculated by dividing the sum volume of CE and TG calculated from the GP-HPLC data by the core volume occupied by CE and TG of one lipoprotein particle.

$${\text{PN}\;\text{for}\;\text{a}\;\text{certain}\;\text{subclass}\;=\;\text{sum}\;\text{of}\;\text{core}\;\text{lipid}\;\text{volume}(\text{V}}_{\text{CE}}{+\;\text{V}}_{\text{TG}}{)/\text{reference}\;\text{core}\;\text{volume}(\text{V}}_{\text{core}})$$

Details of calculation of lipoprotein PNs can be referred to [10] and the patent by Okazaki M. (Publication number: WO/2015/152371).

### Statistical analysis

Data were analyzed by Graphpad Prism Ver.7.01 software program (GraphPad Software, San Diego, CA) and are expressed as the mean±SD. A two-tailed, Student’s *t* test was used for comparison. Statistical significance was established at a p value of <0.05.

## Results

### Lipid profile of patients with CETP-D

The lipid profile of healthy subjects and patients with homozygous CETP-D are shown in Table 1. As we reported previously[15], serum HDL-C and apoA-I levels were markedly higher in patients with CETP-D than in healthy subjects. Furthermore, as a characteristic profile of CETP-D, serum apoC-III and apoE levels were also significantly higher. By contrast, serum LDL-C and apoB levels were significantly lower in CETP-D.

**Table 1:** Clinical characteristics and lipid profiles of healthy subjects and CETP-D patients.

	healthy subjects	CETP-D patients	p value
Age (year)	57.6±14.8	56.7±15.5	0.90
Sex (m/f)	4/5	4/5	
TC (mg/dL)	205.7±29.6	313.9±104.9	0.009
TG (mg/dL)	99.7±26.2	109.6±84.2	0.73
HDL-C (mg/dL)	60.0±8.1	162.2±44.9	<0.001
LDL-C (mg/dL)	112.7±21.8	51.4±11.8	<0.001
FC (mg/dL)	50.2±8.6	82.7±24.4	0.002
PL (mg/dL)	227.1±29.1	351.6±107.6	0.004
Apolipoprotein A-I (mg/dL)	152.8±19.6	245.0±59.6	<0.001
Apolipoprotein A-II (mg/dL)	29.9±2.8	39.1±9.9	0.016
Apolipoprotein B (mg/dL)	93.3±16.1	75.3±13.7	0.02
Apolipoprotein C-II (mg/dL)	4.4±1.0	7.6±2.9	0.007
Apolipoprotein C-III (mg/dL)	9.9±2.2	28.2±18.1	0.009
Apolipoprotein E (mg/dL)	4.5±1.0	14.1±5.8	<0.001
CETP mass (μg/mL)	3.1±0.8	<0.1	<0.001

TC, total cholesterol; TG, triglyceride; HDL-C, high density lipoprotein cholesterol; LDL-C, low density lipoprotein cholesterol; FC, free cholesterol; PL, phospholipid. Data are shown as the mean ± SD and statistical significance was calculated by unpaired Student's T test.

### GP-HPLC patterns and average particle diameters

**Fig 1:**
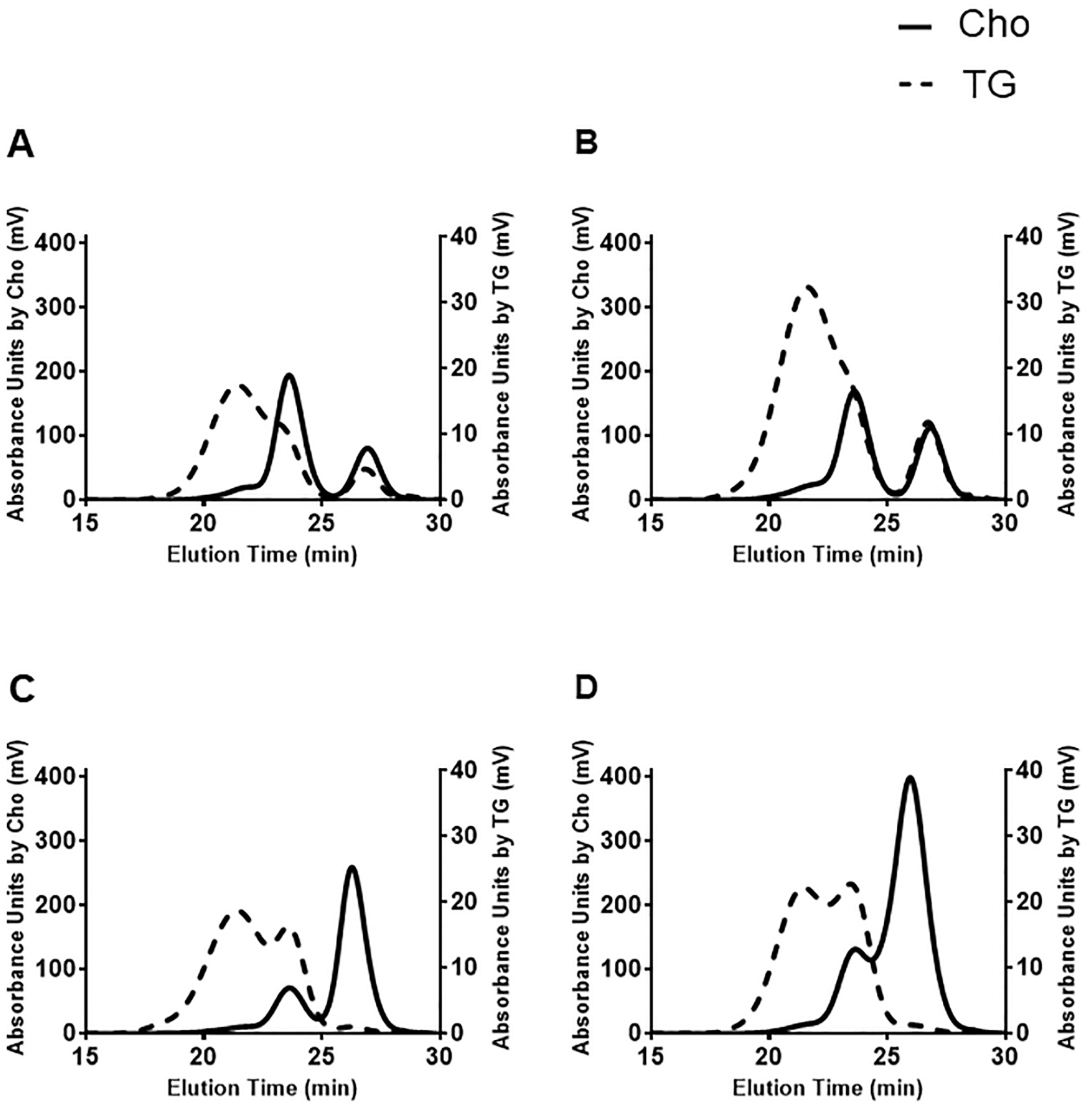
Representative chromatographic patterns of normolipidemic subjects and CETP-D patients monitored by Cho (solid line) and TG (dotted line). (A) a 24-year-old male healthy subject; serum HDL-C: 59mg/dL, LDL-C: 132mg/dL, TG: 83mg/dL. (B) a 52-year-old female healthy subject; serum HDL-C: 69mg/dL, LDL-C: 99mg/dL, TG: 126mg/dL. (C) a 24-year-old male CETP-D patient; serum HDL-C: 138mg/dL, LDL-C: 34mg/dL, TG: 84mg/dL. (D) a 51-year-old female CETP-D patient; serum HDL-C: 197mg/dL, LDL-C: 64mg/dL, TG: 68mg/dL.

The representative chromatographic patterns monitored by Cho (solid line) and TG (dotted line) are shown in Fig 1, including normolipidemic subjects (Fig 1A and 1B) and CETP-D patients (Fig 1C and 1D). In CETP-D, Cho was sparse in the LDL fraction, but was rich in the HDL fraction. By contrast, TG was rich in the LDL fraction, but was scarce in the HDL fraction in patients with CETP-D. Therefore, these data confirmed previous findings that patients with CETP-D have extremely high levels of CE-rich HDL and reduced levels of TG-enriched LDL[15][16].

The average particle diameters of the major lipoprotein classes are shown in Table 2. In the CM -VLDL subclasses, the average particle diameter was not significantly changed between the two groups. In the LDL subclass, the average particle diameter of patients with CETP-D was significantly decreased compared with that of normolipidemic subjects (23.3±1.3 nm vs 25.4±0.2 nm in Cho-monitoring and 25.1±0.3 nm vs 25.9±0.2 nm in TG-monitoring, respectively, p<0.001). By contrast, in the HDL subclass, the average particle diameter of patients with CETP-D was significantly increased compared with that of normolipidemic subjects (12.3±0.4 nm vs 10.7±0.1 nm in Cho-monitoring and 11.6±0.2 nm vs 10.9±0.1 nm in TG-monitoring, respectively, p<0.001), demonstrating that in patients with CETP-D, the LDL particle size is smaller whereas the HDL particle size is larger compared with that of normolipidemic subjects, as we reported previously[15].

**Table 2:** Average particle diameters of lipoprotein major subclass of healthy subjects and CETP-D patients.

	Cho-monitored			TG-monitored		
	healthy subjects	CETP-D patients	p value	healthy subjects	CETP-D patients	p value
CM—VLDL(nm)	39.7±1.4	40.2±2.3	n.p.	44.5±1.6	45.6±2.5	n.p.
LDL (nm)	25.4±0.2	23.3±1.3	p<0.001	25.9±0.2	25.1±0.3	p<0.001
HDL (nm)	10.7±0.1	12.3±0.4	p<0.001	10.9±0.1	11.6±0.2	p<0.001

Data are shown as the mean ± SD and statistical significance was calculated by Student’s T test.

### Cho and TG concentrations in lipoprotein subclasses

**Fig 2:**
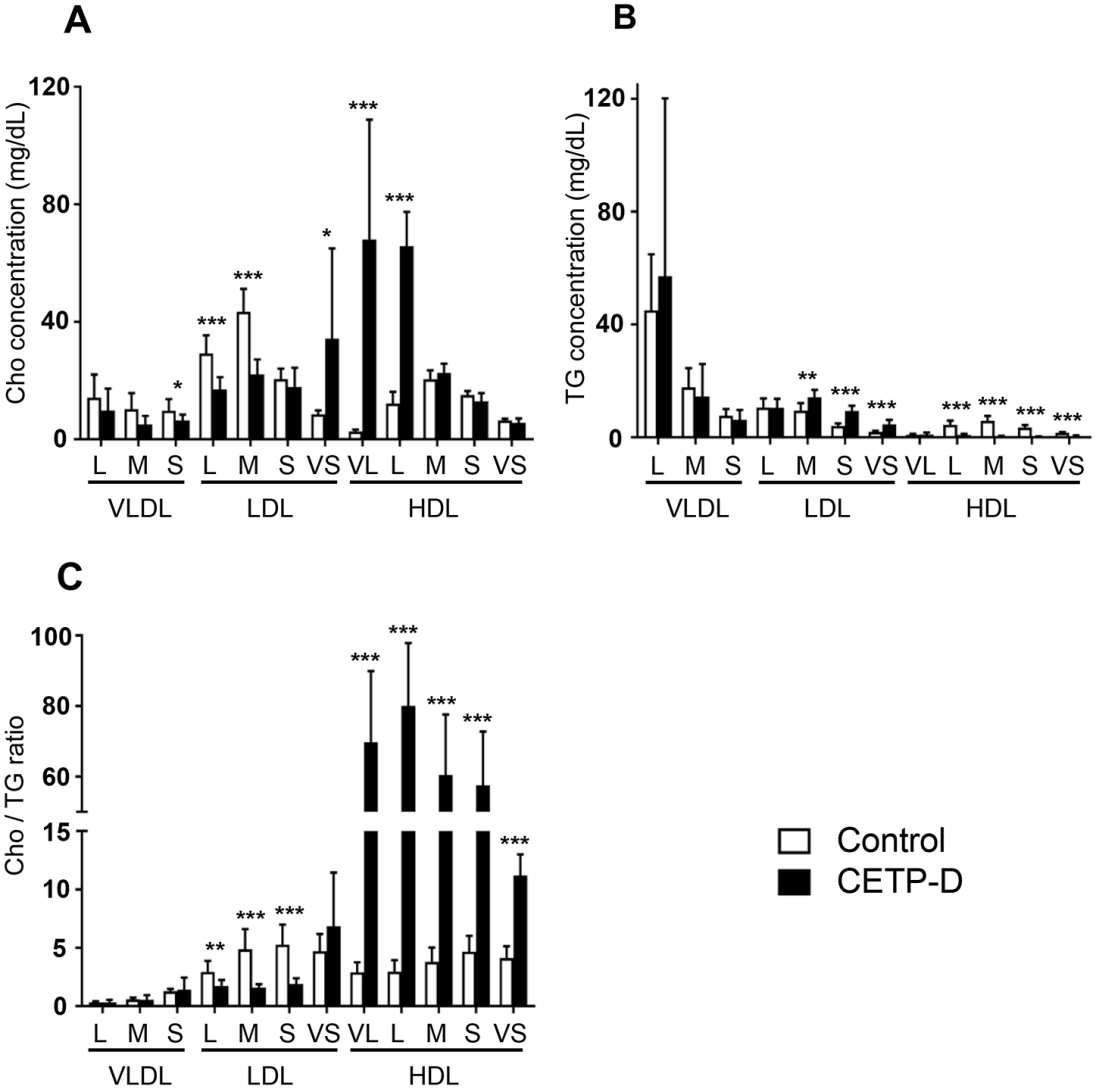
Concentrations of A: Cho (mg/dL), B: TG (mg/dL), C: Cho/TG ratio in lipoprotein subclasses between normolipidemic subjects (n = 9, open column) and CETP-D patients (n = 9, closed column). VL = very large; L = large; M = medium; S = small; VS = very small. All graphs show the mean ± SD. *P<0.05, **P<0.01, ***P<0.001 vs normolipidemic subjects.

The Cho and TG concentrations in lipoprotein subclasses are summarized in Fig 2A–2C. Patients with CETP-D showed a significant decrease in Cho in small VLDL and large to medium LDL subclasses, whereas TG content was unchanged in VLDL and large LDL subclasses or significantly increased in medium to small LDL subclasses. With regard to the Cho/TG ratio, there was no significant change in VLDL subclasses, but the Cho/TG ratios in large to small LDL subclasses were significantly lower, indicating that TG-rich LDL particles are present in CETP-D. In the very small LDL subclass, the Cho and TG content were increased in CETP-D, whereas the Cho/TG ratio was not significantly different between the two groups. However, it should be noted that in the very small LDL subclass, there is also extremely large HDL enriched with apoE in CETP-D[17].

In the HDL fraction, the Cho content was markedly increased in the very large and large HDL subclasses in CETP-D, whereas it was unchanged or slightly decreased in the medium to very small HDL subclasses. By contrast, the TG content was significantly decreased in the large to very small HDL subclasses. The Cho/TG ratios of the HDL subclasses were markedly higher, demonstrating that the HDL particles of CETP-D are more enriched with Cho and more TG depleted.

### Correction for the calculation of the particle number of very, very large HDL (vvl-HDL) in CETP-D

**Fig 3:**
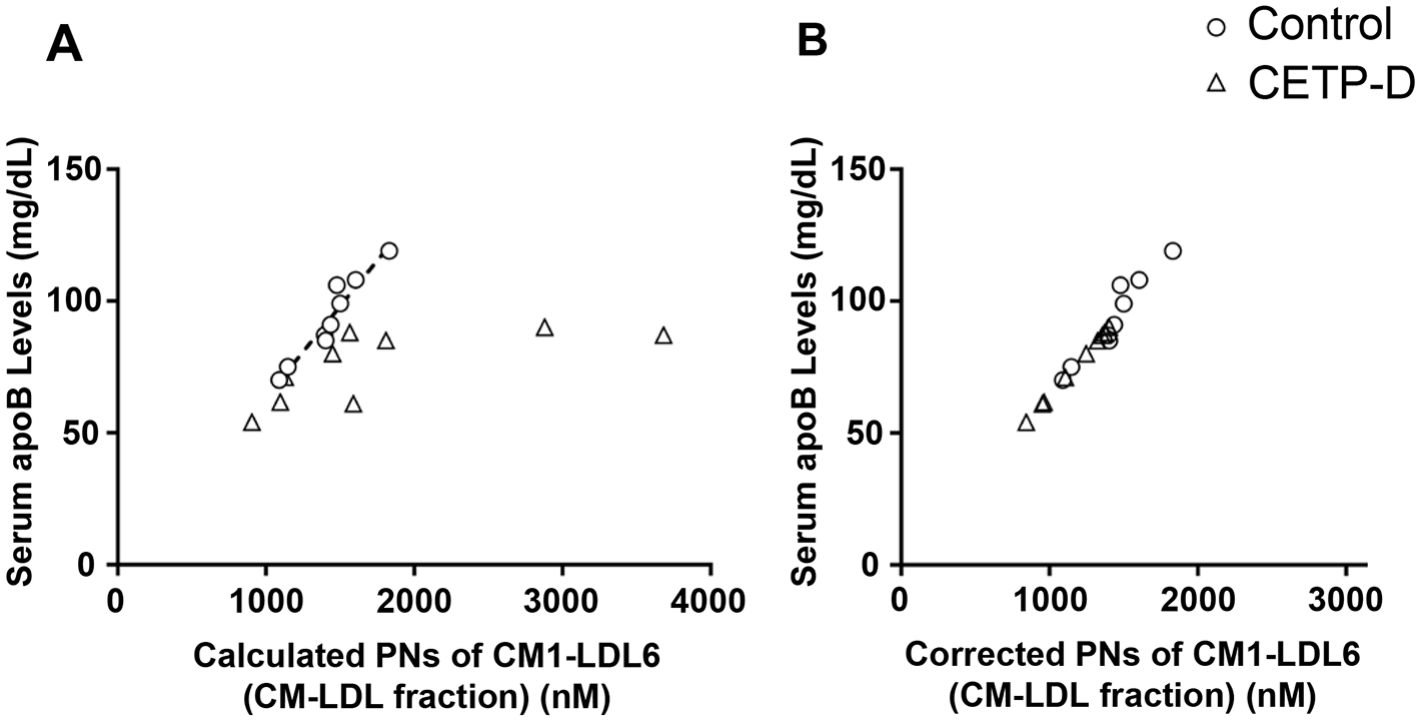
Correction for calculation of the PN of very very large HDL (vvl-HDL) in CETP-D. (A)The relation between the sum of calculated PNs of CM1-LDL6 according to spherical particle model and serum apoB levels in normolipidemic subjects (open circle) and CETP-D patients (open triangle). (B)After correction for PNs of apoB-containing lipoproteins (actual PNs of CM1-LDL6) in normolipidemic subjects (open circle) and CETP-D patients (open triangle).

We calculated the PN of each lipoprotein subclass according to the spherical particle model[10]. In CETP-D, a large amount of extremely large HDL components was eluted between the LDL and HDL positions. We defined the extremely large HDL particle subclasses as very, very large HDL (vvl-HDL), which exists in the very small LDL subclass (LDL4-LDL6). As shown in Fig 3A, the sum of the calculated PNs of CM1-LDL6 according to the spherical particle model (calculated PNs of CM1-LDL6) correlated well with serum apoB levels in normolipidemic subjects (r = 0.92). This result is because one particle of CM1-LDL6 subclasses has just one apoB molecule. By contrast, in CETP-D, the calculated PNs of CM1-LDL6 and serum apoB levels did not show a linear relationship, because there were vvl-HDL particles in the very small LDL fraction. Therefore, in patients with CETP-D, we calculated the PNs of apoB-containing lipoproteins (actual PNs of CM1-LDL6) by multiplying the serum apoB level by 15.6, which is the numeric constant obtained from controls as an average ratio of calculated PNs of CM1-LDL6 (nM) to apoB level (mg/dl) (Fig 3B). The PN of vvl-HDL in patients with CETP-D was calculated by subtracting the calculated PNs of CM1-LDL6 by the actual PNs of CM1-LDL6. Thus, the PN of the very small LDL subclass is calculated by subtracting the sum of the PNs of LDL4-LDL6 by the PN of vvl-HDL.

### Particle numbers of lipoprotein subclasses in CETP-D

**Fig 4:**
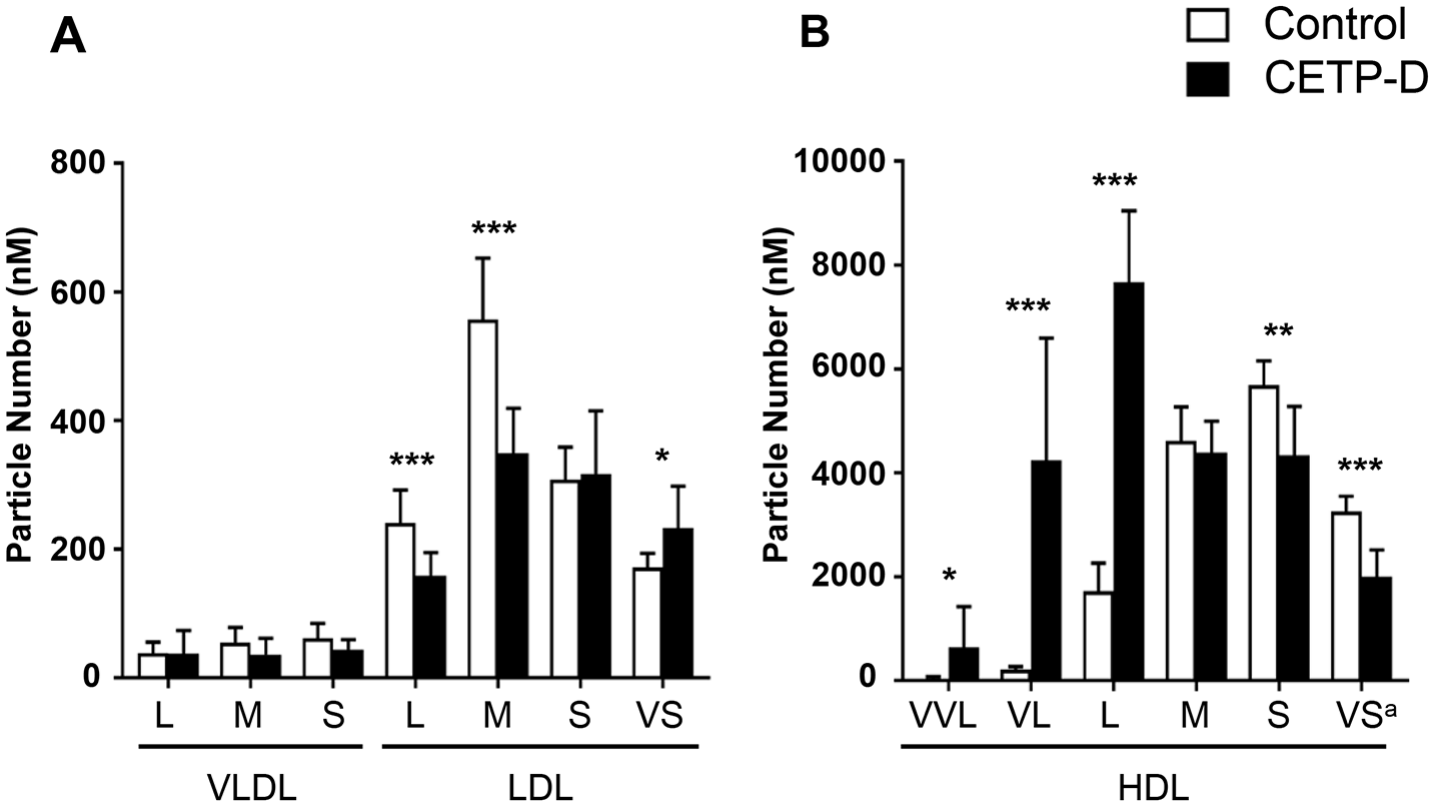
Comparison of particle number of each lipoprotein subclass between normolipidemic subjects (n = 9, open column) and CETP-D patients (n = 9, closed column). (A) VLDL to LDL fraction, (B) HDL fraction. VVL = very very large; VL = very large; L = large; M = medium; S = small; VS = very small. ^a^ PN of very small HDL is assessed only by the PN of HDL6, because HDL7 contains pre-beta-1. HDL for which spherical particle model cannot be applied. All graphs show the mean ± SD. *P<0.05, **P<0.01, *** P<0.001 vs normolipidemic subjects.

As shown in Fig 4, the PN of each VLDL subclass showed no significant difference between normolipidemic subjects and patients with CETP-D. In the LDL fraction, the PNs of large and medium LDL in CETP-D were significantly lower compared with normolipidemic subects (158.3±36.4 nM vs 240.6±51.1 nM, 349.1±69.9 nM vs 557.3±94.8 nM, 0.66- and 0.63-fold decrease, respectively; p<0.001), whereas the PN of very small LDL, which is also termed small dense LDL and known to be atherogenic, was significantly higher (233.2±64.8 nM vs 171.4±22.1 nM, 1.36-fold increase, p = 0.016). In the HDL fraction, very, very large (vvl-)HDL was identified only in CETP-D. The PNs of very large and large HDL were markedly higher in patients with CETP-D (4237.3±2353.4 nM vs 213.4±55.7 nM, 7672.2±1368.3 nM vs 1720.2±536.6 nM, 19.9- and 4.5-fold increase, respectively; p<0.001), whereas the PNs of small and very small HDL were significantly lower (4339.1±937.4 nM vs 5690.3±467.8 nM, 1999.4±514.8 nM vs 3256.5±294.0 nM, 0.76- and 0.61-fold decrease, respectively; p<0.001) compared with normolipidemic subjects. These data suggest that the deficiency of CETP causes marked alterations in the PNs of a variety of LDL and HDL subpopulations in addition to the changes in their lipid composition and size. Interestingly, the increased number of small dense LDL particles may suggest atherogenicity in CETP-D. Furthermore, the marked accumulation of the number of very, very large and very large HDL particles may indicate that apoE and cholesterol-rich HDL particles exhibit delayed catabolism in CETP-D.

## Discussion

We previously reported a case of marked HALP associated with premature corneal opacity and an additional case with coronary artery disease[18]. These cases showed reduced plasma CETP activity, and the composition, size and function of their lipoproteins were abnormal. We also reported that in the Omagari area (Akita Prefecture, Japan), where HALP patients are very frequent, a U-shape relationship was observed between serum HDL-C levels and the incidence of ischemic ECG changes[9], suggesting that patients with HALP due to CETP deficiency were not protected against CVD. Thus, we proposed that HALP is a disorder of reverse cholesterol transport and that CETP-D is an atherogenic HALP[15].

The current study focused on lipoprotein PNs rather than the HDL-C or LDL-C concentration. First, we would like to discuss the accuracy of this GP-HPLC methodology. There have been two primary methods for measuring the PN of HDL[19] other than this GP-HPLC method. The most widely used method is proton nuclear magnetic resonance (NMR) spectrometry[20]. In NMR, lipid methyl groups emit resonances that are unique to the chemical environments of lipoprotein particles of different diameters, with the signal intensity being proportional to their abundance, which makes it possible to quantify not only the PNs of overall HDL but also those of differently sized subspecies. Another method for measuring the PN of HDL is ion mobility (IM) spectrometry, whereby ionized lipoproteins are separated by size and charge in a flow of inert gas subjected to modulated electric fields[21]. After an ultracentrifugation step to remove albumin, the size distributions are presented as PN and mass in eleven lipoprotein subclasses. Although these methods enable the PN measurement of HDL, some issues should be discussed about the accuracy of these methods. The NMR method routinely reports PNs of HDL concentrations to be in the range of 32–34 micro mol/L[22][23], whereas the IM methods reports 5–6 micro mol/L[24][25] in normolipidemic subjects. The NMR data suggest that each HDL particle averages 1.3–1.5 copies of apoA-I. By contrast, the IM data suggest that each HDL particle averages 13–20 copies of apoA-I. This result is apparently discordant with the fact that one HDL particle has 2–5 apoA-I molecules[26]. Calibrated ion mobility (CIM) method, which is an improved method from IM, was recently reported[27]. This CIM method exhibits each HDL particle averages 3–4 copies of apoA-I, in agreement with the current structural model. According to our GP-HPLC and “spherical particle model” method, the average apoA-I molecular number in one HDL particle was 3.062±0.242 in general subjects, as we recently reported[10]. Furthermore, in the same cohort, a very high correlation between the apoB molecular number and the apoB-containing lipoprotein PN was obtained (r = 0.964); an apoB molecule number of 1.041±0.078 was confirmed to be contained per particle, indicating that our methodology seems to be reliable.

A notable finding in the current study is that the PN of larger sized HDL were markedly increased, whereas those of smaller sized HDL were significantly reduced in patients with CETP-D. We have previously reported that large and CE-rich HDL-2 in patients with CETP-D was not able to prevent macrophages from accumulating cholesterol[28]. Furthermore, Gomaraschi M et al.[29] reported that HDL promotes the production of nitric oxide (NO) in endothelial cells, but the HDL and the subfraction from patients with CETP-D was less effective in stimulating NO production, indicating that these larger HDLs are dysfunctional. The increase of larger size HDLs in CETP-D is mainly owing to the disorder of transferring HDL CE to apoB-containing lipoproteins. Another possible mechanism for the increase of larger HDLs may be the delayed catabolism of HDL apoA-I and apoA-II, but not due to the enhanced production rate of these apolipoproteins in CETP-D, as shown by stable isotope studies[30]. In terms of the urinary excretion of HDL particles, smaller HDL-3 filters through the glomerulus into renal tubules. The clearance rate of smaller HDL-3 is regulated by cubilin, which is expressed by absorptive cells, including proximal tubule cells, and mediates the endocytosis of HDL into these cells. By contrast, larger HDL-2 cannot filter through the glomerulus, which results in a different catabolic rate between HDL-2 and HDL-3[31]. The other possible mechanism may be a reduction in the hepatic scavenger receptor BI (SR-BI) in CETP-D. Accordingly, Huang Z et al.[32] previously demonstrated that CETP inhibition leads to reduced hepatic SR-BI *in vitro*. SR-BI is the major receptor for the selective uptake of HDL cholesteryl ester (HDL-CE) and a key receptor for reverse cholesterol transport[33]. The reduction in hepatic SR-BI due to CETP inhibition or deficiency may lead to both impaired selective uptake of SR-BI-mediated HDL-CE and the delayed catabolism of HDL apoA-I. Delayed catabolism of these large HDL particles may result in compositional changes in lipids, proteins, and enzymes associated with HDL, resulting in a variety of alterations in the anti-atherogenic and anti-inflammatory functions of HDL. By contrast, the PN of smaller size HDL particles was significantly reduced in CETP-D. Small HDLs are known to have a potent atheroprotective function, and their concentration is associated with a reduced risk of atherosclerotic diseases[34][35]. The atherogenicity of patients with CETP-D may be attributable to these lipoprotein subclass alterations.

Regarding the LDL fraction, the average particle diameter of patients with CETP-D was 23.3 nm in Cho-monitoring, and 25.1nm in TG-monitoring. This dissociation indicates the polydispersity of LDL in CETP-D, as we reported previously[15]. We also demonstrated that the decreased serum LDL-C level in CETP-D was due to the decreased PNs of large and medium LDL. By contrast, the PN of very small LDL was significantly higher in patients with CETP-D. Very small LDL, namely, small dense LDL, is a small and TG-rich lipoprotein that has a reduced affinity for the LDL receptors of fibroblasts[36] and is well known to be atherogenic due to an increased susceptibility to oxidation[37]. We confirmed the increased PN of this atherogenic very small LDL in patients with CETP-D. Thus, an evaluation of the PNs of LDL subfractions beyond LDL-C would provide additional information about lipoprotein atherogenicity.

Finally, the results of this study for patients with CETP-D may have clinical implications. In patients with CETP-D or those receiving CETP inhibitors, serum HDL-C levels are increased while LDL-C levels are decreased, which seems to be a beneficial lipid profile. However, torcetrapib[5], dalcetrapib[6], and evacetrapib[7] did not demonstrate beneficial cardiovascular outcomes. Anacetrapib has been reported to show a modest reduction of coronary events[8], but the drug development has been discontinued recently. Of course, there may be a large gap between these two conditions, CETP-D and CETP inhibitors plus statins. However, the current extensive analyses of lipoprotein subclasses in CETP-D provide insight into the underlying molecular mechanisms, which help better explain the atherogenic conditions of CETP-D.

There are several limitations in the present study. First, the PNs of chylomicrons or chylomicron remnants cannot be evaluated by this GP-HPLC spherical particle model. This is because chylomicrons are too large, and chylomicron remnants, which have been regarded as an important atherogenic risk factor[38], are as large as VLDL or LDL. The second limitation is that this “spherical particle model” cannot be applied to pre-beta-1 HDL, because pre-beta-1 HDL consists of phospholipids, apoA-I and FC, with no core components. Pre-beta-1 HDL is recognized as the primary acceptor of cholesterol via the ABCA1 transporter. Some studies have shown an association between high levels of this particle and the risk of globally defined coronary artery disease[39]. The trial for the CETP inhibitor evacetrapib was terminated in 2015 because of the absence of beneficial effects on cardiovascular outcomes and increased pre-beta-1 HDL[40]. The third limitation is that we did not analyze the lipoproteins in patients treated with CETP inhibitors, although these two conditions, CETP-D and CETP inhibitors plus statins, show similar lipid profiles. A further investigation of the lipoprotein PN of CETP inhibitor-treated patients should be performed in future studies.

In conclusion, the analysis of lipoprotein PNs in the subclasses of patients with CETP-D has revealed that the PN of larger HDL was markedly increased, that of smaller HDL was decreased, and that of very small LDL was increased in patients with CETP-D, suggesting that CETP-D may have pro-atherogenic lipoprotein properties.

## References

[1] Sharrett AR, Ballantyne CM, Coady SA, Heiss G, Sorlie PD, Catellier D, et al Coronary heart disease prediction from lipoprotein cholesterol levels, triglycerides, lipoprotein(a), apolipoproteins A-I and B, and HDL density subfractions: The Atherosclerosis Risk in Communities (ARIC) Study. Circulation. 2001;104: 1108–13. doi: 10.1161/hc3501.095214 11535564 10.1161/hc3501.095214

[2] Brugts JJ, Yetgin T, Hoeks SE, Gotto AM, Shepherd J, Westendorp RGJ, et al The benefits of statins in people without established cardiovascular disease but with cardiovascular risk factors: meta-analysis of randomised controlled trials. BMJ. 2009;338: b2376 doi: 10.1136/bmj.b2376 19567909 10.1136/bmj.b2376 PMC2714690

[3] Cholesterol Treatment Trialists’ (CTT) Collaboration, Baigent C, Blackwell L, Emberson J, Holland LE, Reith C, et al Efficacy and safety of more intensive lowering of LDL cholesterol: a meta-analysis of data from 170,000 participants in 26 randomised trials. Lancet (London, England). Elsevier Ltd; 2010;376: 1670–81. doi: 10.1016/S0140-6736(10)61350-5 21067804 10.1016/S0140-6736(10)61350-5 PMC2988224

[4] AIM-HIGH Investigators, Boden WE, Probstfield JL, Anderson T, Chaitman BR, Desvignes-Nickens P, et al Niacin in patients with low HDL cholesterol levels receiving intensive statin therapy. N Engl J Med. 2011;365: 2255–67. doi: 10.1056/NEJMoa1107579 22085343 10.1056/NEJMoa1107579

[5] Nissen SE, Tardif J-C, Nicholls SJ, Revkin JH, Shear CL, Duggan WT, et al Effect of torcetrapib on the progression of coronary atherosclerosis. N Engl J Med. 2007;356: 1304–16. doi: 10.1056/NEJMoa070635 17387129 10.1056/NEJMoa070635

[6] Schwartz GG, Olsson AG, Abt M, Ballantyne CM, Barter PJ, Brumm J, et al Effects of dalcetrapib in patients with a recent acute coronary syndrome. N Engl J Med. 2012;367: 2089–99. doi: 10.1056/NEJMoa1206797 23126252 10.1056/NEJMoa1206797

[7] Lincoff AM, Nicholls SJ, Riesmeyer JS, Barter PJ, Brewer HB, Fox KAA, et al Evacetrapib and Cardiovascular Outcomes in High-Risk Vascular Disease. N Engl J Med. 2017;376: 1933–1942. doi: 10.1056/NEJMoa1609581 28514624 10.1056/NEJMoa1609581

[8] Article O. Effects of Anacetrapib in Patients with Atherosclerotic Vascular Disease. N Engl J Med. 2017; NEJMoa1706444. doi: 10.1056/NEJMoa1706444 28847206 10.1056/NEJMoa1706444

[9] Hirano K, Yamashita S, Nakajima N, Arai T, Maruyama T, Yoshida Y, et al Genetic cholesteryl ester transfer protein deficiency is extremely frequent in the Omagari area of Japan. Marked hyperalphalipoproteinemia caused by CETP gene mutation is not associated with longevity. Arterioscler Thromb Vasc Biol. 1997;17: 1053–9. doi: 10.1161/01.ATV.17.6.1053 9194754 10.1161/01.atv.17.6.1053

[10] Okazaki M, Yamashita S. Recent Advances in Analytical Methods on Lipoprotein Subclasses: Calculation of Particle Numbers from Lipid Levels by Gel Permeation HPLC Using “Spherical Particle Model”. J Oleo Sci. 2016;65: 265–82. doi: 10.5650/jos.ess16020 27041512 10.5650/jos.ess16020

[11] Toshima G, Iwama Y, Kimura F, Matsumoto Y, Miura M. LipoSEARCH^®^; Analytical GP-HPLC method for lipoprotein profiling and its applications. J Biol Macromal. 2013;13: 21–32.

[12] Clouet-Foraison N, Gaie-Levrel F, Gillery P, Delatour V. Advanced lipoprotein testing for cardiovascular diseases risk assessment: a review of the novel approaches in lipoprotein profiling. Clin Chem Lab Med. 2017;2453 doi: 10.1515/cclm-2017-0091 28593877 10.1515/cclm-2017-0091

[13] Okazaki M, Usui S, Ishigami M, Sakai N, Nakamura T, Matsuzawa Y, et al Identification of unique lipoprotein subclasses for visceral obesity by component analysis of cholesterol profile in high-performance liquid chromatography. Arterioscler Thromb Vasc Biol. 2005;25: 578–584. doi: 10.1161/01.ATV.0000155017.60171.88 15637308 10.1161/01.ATV.0000155017.60171.88

[14] Okazaki M, Usui S, Fukui A, Kubota I, Tomoike H. Component analysis of HPLC profiles of unique lipoprotein subclass cholesterols for detection of coronary artery disease. Clin Chem. 2006;52: 2049–2053. doi: 10.1373/clinchem.2006.070094 16990425 10.1373/clinchem.2006.070094

[15] Yamashita S, Maruyama T, Hirano KI, Sakai N, Nakajima N, Matsuzawa Y. Molecular mechanisms, lipoprotein abnormalities and atherogenicity of hyperalphalipoproteinemia. Atherosclerosis. 2000;152: 271–285. doi: 10.1016/S0021-9150(00)00574-8 10998455 10.1016/s0021-9150(00)00574-8

[16] Yamashita S, Matsuzawa Y. Re-evaluation of cholesteryl ester transfer protein function in atherosclerosis based upon genetics and pharmacological manipulation. Curr Opin Lipidol. 2016;27: 459–72. doi: 10.1097/MOL.0000000000000332 27454452 10.1097/MOL.0000000000000332

[17] Yamashita S, Sprecher DL, Sakai N, Matsuzawa Y, Tarui S, Hui DY. Accumulation of Apolipoprotein E-Rich High-Density Lipoproteins in Hyperalphalipoproteinemic Human-Subjects with Plasma Cholesteryl Ester Transfer Protein-Deficiency. J Clin Invest. 1990;86: 688–695. doi: 10.1172/JCI114764 2118552 10.1172/JCI114764 PMC296782

[18] Matsuzawa Y, Yamashita S, Funahashi T, Yamamoto A T S. Selective reduction of cholesterol in HDL2 fraction by probucol in familial hypercholesterolemia and hyper HDL2 cholesterolemia with abnormal cholesteryl ester transferTitle. Am J Cardiol. 1988;62: 66B–72B. 3394656 10.1016/s0002-9149(88)80055-9

[19] Davidson WS. HDL-C vs HDL-P: how changing one letter could make a difference in understanding the role of high-density lipoprotein in disease. Clin Chem. 2014;60: e1–3. doi: 10.1373/clinchem.2014.232769 25281702 10.1373/clinchem.2014.232769

[20] Jeyarajah EJ, Cromwell WC, Otvos JD. Lipoprotein particle analysis by nuclear magnetic resonance spectroscopy. Clin Lab Med. 2006;26: 847–70. doi: 10.1016/j.cll.2006.07.006 17110242 10.1016/j.cll.2006.07.006

[21] Caulfield MP, Li S, Lee G, Blanche PJ, Salameh WA, Benner WH, et al Direct determination of lipoprotein particle sizes and concentrations by ion mobility analysis. Clin Chem. 2008;54: 1307–1316. doi: 10.1373/clinchem.2007.100586 18515257 10.1373/clinchem.2007.100586

[22] Mora S, Glynn RJ, Ridker PM. High-density lipoprotein cholesterol, size, particle number, and residual vascular risk after potent statin therapy. Circulation. 2013;128: 1189–1197. doi: 10.1161/CIRCULATIONAHA.113.002671 24002795 10.1161/CIRCULATIONAHA.113.002671 PMC3807967

[23] El Harchaoui K, Arsenault BJ, Franssen R, Després J-P, Hovingh GK, Stroes ESG, et al High-density lipoprotein particle size and concentration and coronary risk. Ann Intern Med. 2009;150: 84–93. Available: http://www.ncbi.nlm.nih.gov/pubmed/19153411 19153411 10.7326/0003-4819-150-2-200901200-00006

[24] Musunuru K, Orho-Melander M, Caulfield MP, Li S, Salameh WA, Reitz RE, et al Ion mobility analysis of lipoprotein subfractions identifies three independent axes of cardiovascular risk. Arterioscler Thromb Vasc Biol. 2009;29: 1975–1980. doi: 10.1161/ATVBAHA.109.190405 19729614 10.1161/ATVBAHA.109.190405 PMC2772123

[25] Krauss RM, Wojnooski K, Orr J, Geaney JC, Pinto CA, Liu Y, et al Changes in lipoprotein subfraction concentration and composition in healthy individuals treated with the CETP inhibitor anacetrapib. J Lipid Res. 2012;53: 540–547. doi: 10.1194/jlr.M018010 22180633 10.1194/jlr.M018010 PMC3276477

[26] Huang R, Silva RAGD, Jerome WG, Kontush A, Chapman MJ, Curtiss LK, et al Apolipoprotein A-I structural organization in high-density lipoproteins isolated from human plasma. Nat Struct Mol Biol. Nature Publishing Group; 2011;18: 416–422. doi: 10.1038/nsmb.2028 21399642 10.1038/nsmb.2028 PMC3079355

[27] Hutchins PM, Ronsein GE, Monette JS, Pamir N, Wimberger J, He Y, et al Quantification of HDL particle concentration by calibrated ion mobility analysis. Clin Chem. 2014;60: 1393–1401. doi: 10.1373/clinchem.2014.228114 25225166 10.1373/clinchem.2014.228114 PMC4324763

[28] Ishigami M, Yamashita S, Sakai N, Arai T, Hirano K, Hiraoka H, et al Large and cholesteryl ester-rich high-density lipoproteins in cholesteryl ester transfer protein (CETP) deficiency can not protect macrophages from cholesterol accumulation induced by acetylated low-density lipoproteins. J Biochem. 1994;116: 257–262. 7822240 10.1093/oxfordjournals.jbchem.a124516

[29] Gomaraschi M, Ossoli A, Pozzi S, Nilsson P, Cefalù AB, Averna M, et al eNOS Activation by HDL Is Impaired in Genetic CETP Deficiency. PLoS One. 2014;9: e95925 doi: 10.1371/journal.pone.0095925 24830642 10.1371/journal.pone.0095925 PMC4022511

[30] Ikewaki K, Rader DJ, Sakamoto T, Nishiwaki M, Wakimoto N, Schaefer JR, et al Delayed Catabolism of High-Density-Lipoprotein Apolipoprotein-a-I and Apolipoprotein-a-Ii in Human Cholesteryl Ester Transfer Protein-Deficiency. J Clin Invest. 1993;92: 1650–1658. doi: 10.1172/JCI116750 8408618 10.1172/JCI116750 PMC288323

[31] Aseem O, Smith BT, Cooley MA, Wilkerson BA, Argraves KM, Remaley AT, et al Cubilin Maintains Blood Levels of HDL and Albumin. J Am Soc Nephrol. 2014;25: 1028–1036. doi: 10.1681/ASN.2013060671 24357674 10.1681/ASN.2013060671 PMC4005305

[32] Huang Z, Inazu A, Kawashiri M, Nohara A, Higashikata T, Mabuchi H. Dual effects on HDL metabolism by cholesteryl ester transfer protein inhibition in HepG2 cells. Am J Physiol Endocrinol Metab. 2003;284: E1210–E1219. doi: 10.1152/ajpendo.00453.2002 12604506 10.1152/ajpendo.00453.2002

[33] Zanoni P, Khetarpal SA, Larach DB, Hancock-Cerutti WF, Millar JS, Cuchel M, et al Rare variant in scavenger receptor BI raises HDL cholesterol and increases risk of coronary heart disease. Science. 2016;351: 1166–71. doi: 10.1126/science.aad3517 26965621 10.1126/science.aad3517 PMC4889017

[34] Chei C-L, Yamagishi K, Kitamura A, Kiyama M, Imano H, Ohira T, et al High-density lipoprotein subclasses and risk of stroke and its subtypes in Japanese population: the Circulatory Risk in Communities Study. Stroke. 2013;44: 327–33. doi: 10.1161/STROKEAHA.112.674812 23321451 10.1161/STROKEAHA.112.674812

[35] Tiozzo E, Gardener H, Hudson BI, Dong C, Della-Morte D, Crisby M, et al High-density lipoprotein subfractions and carotid plaque: The Northern Manhattan Study. Atherosclerosis. Elsevier Ltd; 2014;237: 163–168. doi: 10.1016/j.atherosclerosis.2014.09.002 25240111 10.1016/j.atherosclerosis.2014.09.002 PMC4890152

[36] Sakai N, Yamashita S, Hirano K, Ishigami M, Arai T, Kobayashi K, et al Decreased affinity of low density lipoprotein (LDL) particles for LDL receptors in patients with cholesteryl ester transfer protein deficiency. Eur J Clin Invest. 1995;25: 332–9. Available: http://www.ncbi.nlm.nih.gov/pubmed/7628520 7628520 10.1111/j.1365-2362.1995.tb01710.x

[37] de Graaf J, Hak-Lemmers HL, Hectors MP, Demacker PN, Hendriks JC, Stalenhoef a F. Enhanced susceptibility to in vitro oxidation of the dense low density lipoprotein subfraction in healthy subjects. Arterioscler Thromb. 1991;11: 298–306. doi: 10.1161/01.ATV.11.2.298 1998647 10.1161/01.atv.11.2.298

[38] Masuda D, Yamashita S. Postprandial Hyperlipidemia and Remnant Lipoproteins. J Atheroscler Thromb. 2017;24: 95–109. doi: 10.5551/jat.RV16003 27829582 10.5551/jat.RV16003 PMC5305681

[39] Kane JP, Malloy MJ. Prebeta-1 HDL and coronary heart disease. Curr Opin Lipidol. 2012;23: 367–371. doi: 10.1097/MOL.0b013e328353eef1 22517613 10.1097/MOL.0b013e328353eef1

[40] Nicholls SJ, Ruotolo G, Brewer HB, Kane JP, Wang MD, Krueger KA, et al Cholesterol efflux capacity and pre-beta-1 HDL concentrations are increased in dyslipidemic patients treated with evacetrapib. J Am Coll Cardiol. 2015;66: 2201–2210. doi: 10.1016/j.jacc.2015.09.013 26564598 10.1016/j.jacc.2015.09.013

